# Food Contact Materials: Migration and Analysis. Challenges and Limitations on Identification and Quantification

**DOI:** 10.3390/molecules26113232

**Published:** 2021-05-27

**Authors:** Emmanouil D. Tsochatzis

**Affiliations:** 1 Department of Food Science, Aarhus University, Agro Food Park 48, 8200 Aarhus, Denmark; Emmanouil.tsochatzis@food.au.dk; Tel.: +45-41893130; 2 CiFOOD, Aarhus University Centre for Innovative Food Research, 8200 Aarhus, Denmark

Food contact materials (FCM) are defined as the objects and materials intended to come into direct or indirect contact with foodstuff, while food contact articles are defined as objects, being equipment, containers, packaging and various utensils which are clearly intended to be used for the manufacture, preparation, conservation, flow, transport or handling of foodstuffs [1,2]. Plastic materials are widely used in food packaging applications; however, there is an increasing concern because of the possible release of undesirable components into foodstuffs. During the production of these plastics, several compounds can occur, either intentionally added substances (IAS) like monomers and production chemicals (i.e., antioxidants) or the so-called non-intentionally added substances (NIAS), which are generated as a result of reaction and degradation processes or due to the presence of impurities in the raw materials used for the packaging production. This category of substances might include up to several thousands of compounds, from which the vast majority is unknown. Safety could be threatened by this potentially large number of unknown substances occurring in virgin, processed or recycled plastic FCM. Consequently, concerns have been raised among research communities worldwide regarding FCMs safety and possible adverse health effects on human health, given their abundant presence.

It should be stressed out that although for IAS there is an extensive list of regulated substances and mixtures, according to Commission Regulation (EU) No 10/2011, out of ca. 1000 substances, only for 60% of them analytical standards are available (commercially available or with known purity) and even more a limited number of mass spectral data are available and implemented to analytical databases or libraries.

In the case of NIAS, the situation is even more difficult, as these compounds represent a group of a wide range of compounds, with unknown chemical structure and with no mass spectra data or libraries, whilst in most cases a de novo chemical identification and structural elucidation is required. The aforementioned gives a clear indication of the challenges and limitations for their proper prevalence estimation [1,3].

All the aforementioned are of significant importance, as recycling of plastics for recycled FCM production is nowadays more crucial than ever. Scientific findings showing that FCM are a relevant exposure pathway for the IAS as well as for the plethora of toxicologically uncharacterized NIAS and the fact that these chemical substances are migrating into food. The analytical limitations which burden the evaluation of the prevalence of these substances in food materials are critical and should be addressed using proper tools and knowledge, which are now non-existing.

A representative example of NIAS are the polyester cyclic oligomers which are a ubiquitous category of compounds constituted by multiple repetitions of monomers (primary products) generated during plastic FCM production. Polyester cyclic oligomers have been identified in food simulants and extracts from FCMs, ranging from linear to cyclic structures, presenting a diverse spectrum of physico-chemical properties, e.g. molecular mass, octanol-water partition coefficient (logK_ow_) [4,5,6]. The lack of commercially available oligomer standards, and the lack of mass spectra libraries are a trivial downfall to this direction, as it hinders their confident analytical identification and quantification into food, food simulants or FCMs [3,5,7]. The presence of these substances into virgin and recycled plastics, along with their associated toxicity could subsequently result to an ineffective risk assessment of these FCM. However, society and policies are pushing towards waste reduction with a focus on reuse, recycling or alternative (non-plastic; bio-based) materials production, although in most of the cases the critical aspect of chemical safety, of the individual substances or chemical mixtures, might be critical, especially in case of NIAS.

A comprehensive and robust compound annotation workflow comprises the determination of both volatile and non-volatile compounds, using different chromatographic techniques of diverse selectivity and resolution capacity. However, restricted access to information about the NIAS content present in FCMs, in addition to scarcity in mass spectral data and databases, frame the identification of NIAS substances as a challenging task, despite the advanced and prominent capacity of high resolution mass spectrometry (HR-MS) techniques [5,8]. Even with current state-of-the-art HR-MS, this process involving structural identification and elucidation is very intensive and laborious. Apart from the fact that during identification researchers deal with a large number of non-identified substances, appropriate databases are needed, which are non-existing, although only a fraction of them may be relevant, as in case of NIAS. Finally, it should be mentioned that the different instrumental conditions (e.g., ionization, type of HR-MS) among the different manufacturers makes even more challenging the aforementioned tasks and goals.

Hence, based on the aforementioned, there is no doubt about the scale of the challenges for the chemical identification and structural elucidation of these substances, especially NIAS. This process depends on a number of factors, such as the compound properties, existing concentration in the material (C_p0_) and the availability of mass spectra data, especially coming from high-resolution mass spectrometry [7,9]. In addition to these facts, the existence of different brands of analytical instruments coupled to high-resolution mass spectrometry, making difficult the development of a harmonized strategy or the use of universal libraries. Even more challenging, can be considered the quantification of NIAS due to the lack of analytical standards [5,9,10] making impossible their quantitative analysis. Hence, semi-quantitative strategies are inevitable using internal standards or structural related internal standards [5,11].

Safety requirements for an increasing number of chemicals existing in FCM are highlighting and triggering the need for an efficient screening strategy to prioritize the substances of highest concern and even more the harmonization of chemical analysis, identification, elucidation and quantification of multiple compounds from food contact materials. The latter requires the combination of hyphenated techniques in combination with HR-MS together with the development, integration and use of appropriate mass spectral databases, together with quantitative or semi-quantitative strategies. The latter can be considered as very important, either in cases of IAS or NIAS, where no mass spectra databases are available, nor analytical standards or compounds with a known purity are available. However, the existences of different analytical technologies, especially for the various HR-MS techniques attached to UHPLC systems, makes even more critical and important the implementation and integration of harmonized, validated and inter/instrumental analytical strategies, either for quantification (e.g., semi-quantification) or identification. Finally, the development of proper universal databases and libraries is highly needed to support these harmonized analytical strategies.

Overall, this special issue in *Molecules* focuses on all the aforementioned challenges and will highlight existing analytical approaches, new trends, research work and perspectives.
